# Tigecycline-based versus sulbactam-based treatment for pneumonia involving multidrug-resistant *Acinetobacter calcoaceticus-Acinetobacter baumannii* complex

**DOI:** 10.1186/s12879-016-1717-6

**Published:** 2016-08-05

**Authors:** Jung-Jr Ye, Huang-Shen Lin, Chun-Fu Yeh, Yen-Mu Wu, Po-Yen Huang, Chien-Chang Yang, Ching-Tai Huang, Ming-Hsun Lee

**Affiliations:** 1 Division of Infectious Diseases, Department of Internal Medicine, Chang Gung Memorial Hospital at Linkou, Chang Gung University College of Medicine, 5 Fu-Shin St., Gueishan 333 Taoyuan, Taiwan; 2 Division of Infectious Diseases, Department of Internal Medicine, Chang Gung Memorial Hospital, Cha-Yi, Taiwan

## Abstract

**Background:**

The treatment options for pneumonia involving multidrug-resistant *Acinetobacter calcoaceticus-Acinetobacter baumannii* (MDR Acb) complex are limited, and the optimal treatment has not been established.

**Methods:**

To compare the efficacy of tigecycline-based with sulbactam (or ampicillin/sulbactam)-based therapy for pneumonia involving MDR Acb complex, we conducted a retrospective study comparing 84 tigecycline-treated adult patients during the period August 2007 to March 2010 with 84 sulbactam or ampicillin/sulbactam-treated adult patients during the period September 2004 to July 2007. Both groups had the matched Acute Physiology and Chronic Health Evaluation (APACHE) II score and received treatment for at least 7 days.

**Results:**

The mean APACHE II score was 20.1 for both groups. More patients in sulbactam group had ventilator use (89.3 % versus 69.0 %), bilateral pneumonia (79.8 % versus 60.7 %) and combination therapy (84.5 % versus 53.6 %), particularly with carbapenems (71.4 % versus 6.0 %), while more patients in tigecycline group had delayed treatment (41.7 % versus 26.2 %) (*P* <0.05). At the end of treatment, more patients in sulbactam group had airway MDR Acb complex eradication (63.5 % versus 33.3 %, *P* <0.05). The clinical resolution rate was 66.7 % for both groups. The mortality rate during treatment was 17.9 % in sulbactam group, and 25.0 % in tigecycline group (*P* = 0.259). The multivariate analysis showed that bilateral pneumonia was the only independent predictor for mortality during treatment (adjusted odds ratio, 2.717; 95 % confidence interval, 1.015 to 7.272).

**Conclusions:**

Patients treated with either tigecycline-based or sulbactam-based therapy had a similar clinical outcome, but tigecycline group had a lower microbiological eradiation rate.

## Background

Pneumonia involving multidrug-resistant (MDR) *Acinetobacter calcoaceticus-Acinetobacter baumannii* (Acb) complex usually occurs in critically ill patients and is associated with unfavorable outcomes [1–3]. For MDR Acb complex resistant to most currently available antibiotics, including β-lactams, fluoroquinolones, and aminoglycosides, there are only a few treatment options, such as tigecycline, sulbactam, and colistin [4, 5].

Tigecycline is a glycylcycline with in vitro activity against MDR Acb complex [6]. The comparison analysis from the U.S. Food and Drug Administration showed that tigecycline treatment had a higher mortality rate than other antimicrobials in ventilator associated pneumonia (VAP) [7]. A recent study also reported a significantly lower cure rate in clinically evaluable patients with VAP treated with tigecycline when compared to imipenem (47.9 % versus 70.1 %) [8]. However, for pneumonia caused by MDR Acb complex resistant to carbapenems and other classes of antibiotics, off label use of tigecycline was common in clinical practice, and the clinical response rates ranged from 60 to 88 % in prior studies [9–11]. Sulbactam is a β-lactamase inhibitor with antimicrobial activity against *Acinetobacter* species [12]. It is available alone or in combination with ampicillin, and ampicillin doesn’t contribute activity or synergism against *A. baumannii* [12]. Sulbactam or ampicillin/sulbactam had clinical response rates ranging from 67 to 75 % for pneumonia involving MDR *A. baumannii* (MDRAB) or MDR Acb complex in prior studies [13–15].

In our hospital, tigecycline was not available until August 2007. Before that, sulbactam or ampicillin/sulbactam might be the only treatment option with in vitro activity against MDR Acb complex. Thus, we conducted a retrospective study to compare the efficacy of tigecycline-based with sulbactam (or ampicillin/sulbactam)-based treatment for pneumonia involving MDR Acb complex. With a match in the Acute Physiology and Chronic Health Evaluation (APACHE) II score for both groups, a comparison was made between tigecycline-treated adult patients during the period August 2007 to March 2010 and sulbactam (or ampicillin/sulbactam)-treated adult patients during the period September 2004 to July 2007. The clinical efficacy, outcomes and microbiological eradication were included for analyses.

## Methods

### Setting

Chang Gung Memorial Hospital (CGMH)-Linkou is a university-affiliated medical center providing both primary and tertiary health care in northern Taiwan. This retrospective study has been approved by institutional review boards of CGMH- Linkou (Number: 99-1478B and 100-0294B). The ethics committee granted a waiver for informed consent to be obtained.

### Study design, patients and treatments

All hospitalized patients who were ≧ 18 years old and had pneumonia involving MDR Acb complex treated with tigecycline between August 2007 and March 2010, and sulbactam or ampicillin/sulbactam between September 2004 and July 2007, were reviewed. Each tigecycline-treated patient was matched to one sulbactam or ampicillin/sulbactam-treated patient based on identical values of APACHE II score and chart number sequence. Patients were excluded if they did not have a matched control or had a combination therapy with tigecycline and sulbactam (or ampicillin/sulbactam). Patients with initial bacteremia were also excluded since tigecycline treatment for bacteremia was controversial.

Pneumonia was diagnosed if the patient had a radiographic infiltrate that was new or progressive, along with at least two of the following clinical characteristics: new onset of fever (≧ 38 °C) or hypothermia (< 35.5 °C), leucocytosis (leucocyte count > 12000 cells/mm^3^) or leucopenia (leucocyte count < 4000 cells/mm^3^), decline in oxygenation (O2 saturation < 90 %), and increasing amount of purulent sputum [16]. Pneumonia involving MDR Acb complex was defined as clinical evidence of pneumonia with sputum or tracheal aspirate cultures positive for MDR Acb complex from 1 week before to 3 days after the first dose of tigecycline or sulbactam or ampicillin/sulbactam. Tracheal aspirate and sputum specimens were sent for bacterial culture only if their Gram’s stains showed at least 25 neutrophils and less than 10 epithelial cells per low-power field. Growth was assessed semi-quantitatively. The etiologic pathogen of pneumonia was determined if the tracheal aspirate or sputum culture had an at least moderate growth, i.e., the growth confined up to primary streaking line and > 5 colonies in secondary streaking zone [17]. Polymicrobial pneumonia was defined as one or more additional etiologic bacterial species concurrently isolated from the respiratory tract during treatment.

All patients in tigecycline group received tigecycline for at least 7 days, with a 100-mg loading dose followed by 50 mg administered intravenously every 12 h. All patients in sulbactam group received intravenous sulbactam 1 g or ampicillin/sulbactam 3 g (at a ratio 2:1) every 6 or 8 h for at least 7 days. Dose and dosing interval were adjusted according to serum creatinine levels. Combination therapy was defined as simultaneous use of another class of antibiotics for at least 3 days. These antibiotics included carbapenems (meropenem or imipenem), fluoroquinolones (ciprofloxacin or levofloxacin), amikacin, cephalosporins (ceftazidime or cefepime), piperacillin, piperacillin-tazobactam, colistimethate, and aztreonam. Delayed treatment was defined as more than 3 days between the detection of airway MDR Acb complex isolates and the first dose of tigecycline or sulbactam or ampicillin/sulbactam.

### Microbiology

Identification of Acb complex depended upon Gram staining and conventional biochemical tests [18]. Briefly, the isolates were identified as species of the genus *Acinetobacter* based on the following properties: aerobic, Gram-negative, nonmotile coccobacillary rods with a nonfermentative, catalase-positive and oxidase-negative reaction. *Acinetobacter* species with glucose-oxidizing non-haemolytic characteristics were classified as Acb complex. Antimicrobial susceptibility was determined and interpreted according to the criteria of Clinical and Laboratory Standards Institute by disk diffusion method [19]. Susceptibility to tigecycline was determined using disk diffusion method with Mueller-Hinton agar (BD Microbiology Systems, Cockeysville, MD) with the resistant breakpoint at ≧ 16 mm and susceptible breakpoint at ≦ 12 mm [20]. An isolate with full or intermediate resistance to amikacin, gentamicin, cefepime, ceftazidime, aztreonam, piperacillin, piperacillin-tazobactam, ciprofloxacin, imipenem and meropenem was defined as MDR Acb complex [21].

Cultures were collected from 1 week before the first dose of tigecycline or sulbactam (or ampicillin/sulbactam) to the discharge of patients. Pathogens, sites of growth and susceptibility testing were recorded. Microbial eradication of MDR Acb complex was defined as no growth of Acb complex or susceptibility change from MDR strains to susceptible strains in Acb complex in follow-up respiratory tract cultures before and 7 days after treatment cessation. Relapse was defined as new isolation of MDR Acb complex from the respiratory tract cultures within 2 weeks after initial eradication. Initial bacteremia was defined as bacteremia at the beginning of treatment, which meant at least one positive blood culture 1 week before to 3 days after the first dose of tigecycline or sulbactam or ampicillin/sulbactam. Bacteremia during treatment was defined as at least one positive blood culture 3 days after to the end of treatment.

### Demography and comorbidity

Data on age, sex, surgery, and co-morbid illness were gathered by reviewing in-patient medical records. Co-morbid illness included hepatic dysfunction of a serum total bilirubin level over 2.5 mg/dL or liver cirrhosis, renal insufficiency of a serum creatinine level above 2.0 mg/dL or requirement of dialysis, chronic pulmonary disease, heart disease, diabetes mellitus, immune compromise, and hematological or solid organ malignancy. Immune compromise was defined by at least one of the following: use of prednisone or equivalent over 20 mg per day for at least 2 weeks, organ transplant recipient, human immunodeficiency virus infection or acquired immunodeficiency syndrome, neutropenia (absolute neutrophil count less than 500 cells/mm3), use of immunosuppressive agents, and concurrent hematological malignancy.

### Clinical conditions and outcomes

Ventilator use, vital signs, and infections other than pneumonia during treatment were recorded. Defervescence was defined as normal body temperature for at least 3 days at the end of treatment. Severity of illness was assessed by a modified APACHE II score, which was recorded within 48 h before or after the first dose of tigecycline or sulbactam or ampicillin/sulbactam. The 30-day mortality was defined as death occurring within 30 days after treatment. The chest radiographs were evaluated by at least two investigators. A series of chest radiographs were evaluated during treatment. Clinical resolution of pneumonia at the end of treatment was defined as (1) decreased pulmonary infiltrate, and (2) survival with stationary findings on chest radiographs and defervescence. Thus, patients with persistent fever or death during treatment would be defined as clinical failure if infiltrates were stationary. Progressing infiltrates were defined as clinical failure.

### Statistical methods

All statistical analyses were performed using the Statistical Package for the Social Sciences for Windows (Version 15.0; SPSS Inc., Chicago, IL, USA). Categorical variables were compared using χ^2^ test or Fisher exact test, as appropriate. Continuous variables were tested for normality of distributions by Kolmogorov–Smirnov test, and then compared by Student’s *t-*test or the Mann-Whitney *U* test, as appropriate. Odds ratios (ORs) and 95 % confidence interval (CI) were calculated. The survival curve was plotted by means of the Kaplan-Meier method, and the log rank test was used to compare univariate survival distribution between tigecycline and sulbactam groups. Variables with a *P* value < 0.1 in univariate analysis and tigecycline use were included in a logistic regression model for multivariate analysis. All tests were two-tailed, and a *P* value of < 0.05 was considered significant.

## Results

### Patients, demography and concomitant diseases

One hundred and sixteen tigecycline-treated episodes of pneumonia involving MDR Acb complex were identified in 112 patients, while 177 sulbactam or ampicllin/sulbactam-treated episodes were identified in 173 patients. Finally, 84 tigecycline-treated patients were enrolled and matched to 84 patients treated with sulbactam (26 patients) or ampicillin/sulbactam (58 patients). The mean APACHE II score was 20.1 for both groups. In tigecycline group, 59 (70.2 %) and 25 (29.8 %) patients had positive MDR Acb complex cultures from tracheal aspirates and sputum, respectively. In sulbactam group, 73 (86.9 %) and 11 (13.1 %) patients had that from tracheal aspirates and sputum, respectively. There was no significant difference in age, gender, and concomitant diseases between the two groups (Table 1).

**Table 1:** The comparison analysis of demography, concomitant diseases, clinical features, and outcomes between tigecycline (TG) and sulbactam (SB) groups

Variables	TG group^a^	SB group^a^	*p*	OR (95 % CI)
	*n* = 84	*n* = 84		
Demographic parameters				
Age, yr	69.6 (15.9)	70.6 (15.6)	0.689	
Male gender	57 (67.9)	58 (69.0)	0.868	0.946 (0.494–1.814)
Concomitant diseases				
Hepatic dysfunction	12 (14.3)	6 (7.1)	0.134	2.167 (0.773–6.075)
Renal insufficiency	32 (38.1)	32 (38.1)	1.000	1.000 (0.536–1.864)
Chronic pulmonary disease	22 (26.2)	20 (23.8)	0.722	1.135 (0.564–2.284)
Heart disease	13 (15.5)	7 (8.3)	0.153	2.014 (0.761–5.333)
Diabetes mellitus	26 (31.0)	35 (41.7)	0.149	0.628 (0.333–1.183)
Immune compromise	13 (15.5)	11 (13.1)	0.659	1.215 (0.511–2.891)
Malignancy	15 (17.9)	20 (23.8)	0.342	0.696 (0.328–1.474)
Surgery	22 (26.2)	15 (17.9)	0.193	1.632 (0.778–3.423)
Clinical conditions				
APACHE II Score	20.1 (6.1)	20.1 (6.1)	1.000	
Ventilator use	58 (69.0)	75 (89.3)	0.001	0.268 (0.117–0.615)
Pneumonia involving bilateral lung	51 (60.7)	67 (79.8)	0.007	0.392 (0.197–0.781)
Polymicrobial pneumonia, overall	66 (78.6)	62 (73.8)	0.469	1.301 (0.638–2.654)
Polymicrobial pneumonia, coinfection with				
MRSA	26 (31.0)	34 (40.5)	0.198	0.659 (0.349–1.245)
*Pseudomonas aeruginosa*	33 (39.3)	23 (27.4)	0.102	1.716 (0.896–3.285)
*Klebsiella* spp*.* ^b^	12 (14.3)	5 (6.0)	0.073	2.633 (0.884–7.840)
*Escherichia coli*	2 (2.4)	2 (2.4)	1.000	1.000 (0.138–7.270)
*Enterobacter* spp*.* ^c^	2 (2.4)	2 (2.4)	1.000	1.000 (0.138–7.270)
*Serratia marcescens*	10 (11.9)	1 (1.2)	0.005	11.216 (1.402–89.724)
*Stenotrophomonas maltophilia*	6 (7.1)	15 (17.9)	0.036	0.354 (0.130–0.962)
Multisite infections, overall	33 (39.3)	35 (41.7)	0.753	0.906 (0.489–1.678)
With urinary tract infection	13 (15.5)	19 (22.6)	0.238	0.626 (0.287–1.369)
With catheter related infection	2 (2.4)	10 (11.9)	0.017	0.180 (0.038–0.851)
With soft tissue and wound infection	10 (11.9)	5 (6.0)	0.176	2.135 (0.697–6.540)
With intra-abdominal infection	8 (9.5)	4 (4.8)	0.231	2.105 (0.609–7.279)
With invasive fungal infection^d^	12 (14.3)	4 (4.8)	0.035	3.333 (1.029–10.799)
Bacteremia during treatment	4 (4.8)	0 (0.0)	0.121	9.447 (0.501–178.291)
With TG or SB-resistant MDR Acb complex^e^	16 (19.0)	43 (51.2)	< 0.0001	0.224 (0.112–0.448)
Treatment				
Duration, days	14.6 (5.4)	16.4 (7.6)	0.150	
Combination therapy, overall	45 (53.6)	71 (84.5)	< 0.0001	0.211 (0.102–0.439)
With cephalosporins	20 (23.8)	8 (9.5)	0.013	2.969 (1.226–7.192)
With colistin	12 (14.3)	0 (0.0)	< 0.0001	29.138 (1.695–500.773)
With carbapenems	5 (6.0)	60 (71.4)	< 0.0001	0.025 (0.009–0.070)
With aminoglycosides	7 (8.3)	1 (1.2)	0.064	7.545 (0.907–62.744)
With fluoroquinolones	12 (14.3)	4 (4.8)	0.035	3.333 (1.029–10.799)
Delayed treatment	35 (41.7)	22 (26.2)	0.034	2.013 (1.049–3.863)
Outcomes				
Airway eradication of MDR Acb complex without relapse^f^	26 (33.3)	47 (63.5)	< 0.0001	0.287 (0.147–0.560)
Defervescence	54 (64.3)	76 (90.5)	< 0.0001	0.189 (0.081–0.445)
Image study of lung				
Improvement	37 (44.0)	39 (46.4)	0.757	0.908 (0.495–1.668)
Stationary	32 (38.1)	22 (26.2)	0.099	1.734 (0.900–3.342)
Deterioration	15 (17.9)	23 (27.4)	0.140	0.577 (0.276–1.204)
Clinical resolution of pneumonia	56 (66.7)	56 (66.7)	1.000	1.000 (0.526–1.899)
Mortality during treatment	21 (25.0)	15 (17.9)	0.259	1.533 (0.728–3.231)
30-day mortality	28 (33.3)	25 (29.8)	0.618	1.180 (0.615–2.264)

*Abbreviations*: *TG* tigecycline, *SB* sulbactam, *OR* odd ratio, *CI* confidence interval, *APACHE* acute physiology and chronic health evaluation, *MRSA* methicillin resistant *Staphylococcus aureus*, *MDR Acb* multidrug resistant *Acinetobacter calcoaceticus-Acinetobacter baumannii*, *ESBL* extended-spectrum beta-lactamase

^a^Categorical data are no. (%) of subject, continuous data are expressed as mean (standard deviation)

^b^16 patients had coinfection with *Klebsiella pneumoniae*, including 10 with ESBL strains, and 1 had *Klebsiella oxytoca*-ESBL

^c^3 patients had coinfection with *Enterobacter cloacae*, and 1 had *Enterobacter aerogenes*

^d^15 patients had candidemia, and 1 had possible invasive aspergillosis diagnosed with positive serum galactomannan

^e^With TG-resistant MDR Acb complex during TG treatment in TG group, or with SB-resistant MDR Acb commplex during SB treatment in SB group

^f^78 patients in TG group and 74 in SB group had available data for evaluation

### Clinical conditions

Patients in sulbactam group had more ventilator use (89.3 % versus 69.0 %) and bilateral pneumonia (79.8 % versus 60.7 %) than those in tigecycline group. There were no significant differences between these two groups in the overall rates of polymicrobial pneumonia and multisite infections. *Pseudomonas aeruginosa* and *Methicillin-resistant Staphylococcus aureus* were the most common concurrent pathogens for pneumonia, and urinary tract infection was the most common concurrent infection. However, more patients had *Serratia marcescnes* coinfection and invasive fungal infection in tigecycline group, and more patients had *Stenotrophomonas maltophilia* coinfection and catheter related infection in sulbactam group. Among the 168 enrolled patients, bacteremia during treatment was observed in four patients, and all of them were from tigecycline group (*P* = 0.121). During treatment, tigecycline-resistant MDR Acb complex was isolated in 16 (19.0 %) tigecycline-treated patients, and sulbactam-resistant MDR Acb complex was isolated in 43 (51.2 %) sulbactam or ampicllin/sulbactam-treated patients (19.0 % versus 51.2 %, *P* < 0.0001) (Table 1). In tigecycline group, 71 patients (84.5 %) had airway MDR Acb complex isolates with full or intermediate resistance to sulbactam. Tigecycline susceptibility testing was not performed in sulbactam group.

### Treatment

The mean treatment duration was 14.6 and 16.4 days for tigecycline and sulbactam group, respectively. Compared to the tigecycline group, more patients in sulbactam group had combination therapy (84.5 % versus 53.6 %), particularly with carbapenems (71.4 % versus 6.0 %). In sulbactam group, the most common co-administered agent were carbapenems (60/71, 84.5 %), followed by cephalosporins (8/71, 11.3 %), and 32 patients (38.1 %) had glycopeptides use. In tigecycline group, the most common co-administered agent was cephalosporins (20/45, 44.4 %), followed by colistin (12/45, 26.7 %) and fluoroquinolones (12/45, 26.7 %). Colistin was not available until May 2007 in our hospital, and co-use of colistin was only noted in tigecycline group. More patients in tigecycline group had delayed treatment (41.7 % versus 26.2 %) (Table 1).

### Outcomes

**Fig. 1:**
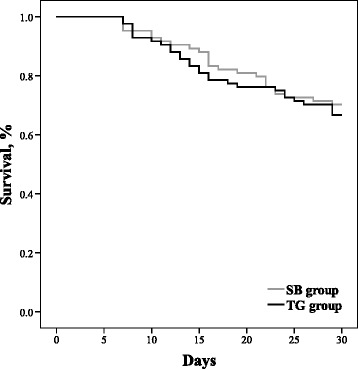
Comparative survival curves for tigecycline (*black line*) and sulbactam (*gray line*) groups; Log-rank test: *p* = 0.605. *Abbreviations*: *SB* sulbactam, *TG* tigecycline

Sulbactam group had a higher rate of airway MDR Acb complex eradication (63.5 % versus 33.3 %) and defervescence (90.5 % versus 64.3 %) than tigecycline group at the end of treatment. There was no significant difference between these two groups in the rates of clinical resolution, 30-day mortality and mortality during treatment (66.7 % versus 66.7 %; 33.3 % versus 29.8 %; 25.0 % versus 17.9 %, respectively) (Table 1). The cumulative survival rate at 30 days was similar between the two groups by Kaplan-Meier method (Fig. 1).

### The predictor for mortality during treatment

In the univariate analysis, the survivors were more likely to have tigecycline or sulbactam-resistant MDR Acb complex than the deceased (39.4 % versus 19.4 %) (Table 2). And most of the resistant isolates in survivors were from sulbactam group (39/52, 75 %). In the multivariate analysis including tigecycline-based treatment and variables with a *P* value < 0.1 in the univariate analysis, bilateral pneumonia was the only independent predictor for mortality during treatment (adjusted OR, 2.717; 95 % CI, 1.015 to 7.272) (Table 2). Other models of multivariate analysis including polymicrobial pneumonia, combination therapy, and combination with carbapenem or colistin also showed that bilateral pneumonia was the only independent predictor (Table 3).

**Table 2:** Univariate and multivariate analyses of the predictors for mortality during treatment of tigecycline or sulbactam or ampicillin/sulbactam for pneumonia involving multidrug resistant *Acinetobacter calcoaceticus-Acinetobacter baumannii* (MDR Acb) complex

Variables	Deceased^a^	Survivors^a^	Univariate	Multivariate^b^	
	*n* = 36	*n* = 132	*p*	*p*	Adjusted OR (95 % CI)
Demographic parameters					
Age, yr	69.8 (14.7)	70.2 (16.0)	0.658		
Male gender	26 (72.2)	89 (67.4)	0.583		
Concomitant diseases					
Hepatic dysfunction	6 (16.7)	12 (9.1)	0.224		
Renal insufficiency	15 (41.7)	49 (37.1)	0.619		
Chronic pulmonary disease	6 (16.7)	36 (27.3)	0.193		
Heart disease	6 (16.7)	14 (10.6)	0.383		
Diabetes mellitus	14 (38.9)	47 (35.6)	0.717		
Immune compromise	5 (13.9)	19 (14.4)	0.939		
Malignancy	11 (30.6)	24 (18.2)	0.105		
Surgery	11 (30.6)	26 (19.7)	0.163		
Clinical conditions					
APACHE II Score	20.3 (7.3)	20.1 (5.8)	0.858		
Ventilator use	26 (72.2)	107 (81.1)	0.247		
Bilateral pneumonia	30 (83.3)	88 (66.7)	0.053	0.047	2.717 (1.015–7.272)
Polymicrobial pneumonia	29 (80.6)	99 (75.0)	0.488		
With MRSA	10 (27.8)	50 (37.9)	0.262		
With *Pseudomonas aeruginosa*	14 (38.9)	42 (31.8)	0.425		
With *Klebsiella* spp.	6 (16.7)	11 (8.3)	0.207		
With *Serratia marcescens*	3 (8.3)	8 (6.1)	0.704		
With *Stenotrophomanas maltophilia*	6 (16.7)	15 (11.4)	0.400		
Multisite infections	18 (50.0)	50 (37.9)	0.189		
With urinary tract infection	8 (22.2)	24 (18.2)	0.584		
With catheter related infection	2 (5.6)	10 (7.6)	1.000		
With skin and soft tissue infection	6 (16.7)	9 (6.8)	0.094	0.214	2.070 (0.657–6.521)
With intra-abdominal infection	3 (8.3)	9 (6.8)	0.721		
With invasive fungal infection	3 (8.3)	13 (9.8)	1.000		
Microbiology					
MDR Acb complex with TG or SB resistance^c^	7 (19.4)	52 (39.4)	0.026	0.079	0.426 (0.164–1.103)
Airway eradication of MDR Acb complex^d^	11 (42.3)	62 (49.2)	0.521		
Bacteremia during treatment	2 (5.6)	2 (1.5)	0.201		
Treatment					
Tigecycline-based treatment	21 (58.3)	63 (47.7)	0.259	0.451	1.371 (0.604–3.116)
Duration, days	15.6 (7.9)	15.5 (6.2)	0.519		
Combination therapy	25 (69.4)	91 (68.9)	0.954		
With cephalosporins	6 (16.7)	22 (16.7)	1.000		
With carbapenems	12 (33.3)	53 (40.2)	0.457		
With fluoroquinolones	2 (5.6)	14 (10.6)	0.527		
With colistin	3 (8.3)	9 (6.8)	0.721		
Delayed treatment	9 (25.0)	48 (36.4)	0.202		

*Abbreviations*: *MDR Acb* multidrug resistant *Acinetobacter calcoaceticus-Acinetobacter baumannii*, *OR* odd ratio, *CI* confidence interval, *APACHE* acute physiology and chronic health evaluation, *MRSA* methicillin resistant *Staphylococcus aureus*, *TG* tigecycline, *SB* sulbactam

^a^Categorical data are no. (%) of subject, continuous data are expressed as mean (standard deviation)

^b^All variables included in the final multivariable model are shown

^c^ The initial airway MDR Acb complex isolates with resistance to TG in TG group, or with resistance to SB in SB group

^d^26 patients in the deceased group and 126 in the survivors group had available data for evaluation

**Table 3:** Multivariate analyses of the predictors for mortality during treatment including combination therapy, carbapenems or colistin use, and polymicrobial pneumonia

Variables	Odds ratio	95 % confidence interval	*p*
Model A^a^			
With skin and soft tissue infection	2.041	0.644–6.466	0.225
MDR Acb complex with TG or SB resistance	0.418	0.160–1.092	0.075
Bilateral pneumonia	2.663	0.987–7.186	0.053
Tigecycline-based treatment	1.405	0.608–3.245	0.426
Combination therapy	1.133	0.472–2.720	0.779
Model B^b^			
With skin and soft tissue infection	2.071	0.657–6.523	0.214
MDR Acb complex with TG or SB resistance	0.426	0.163–1.114	0.082
Bilateral pneumonia	2.717	1.007–7.329	0.048
Tigecycline-based treatment	1.373	0.497–3.795	0.541
Combination with carbapenem	1.002	0.346–2.905	0.997
Model C^c^			
With skin and soft tissue infection	2.002	0.624–6.425	0.243
MDR Acb complex with TG or SB resistance	0.420	0.161–1.090	0.075
Bilateral pneumonia	2.795	1.028–7.600	0.044
Tigecycline-based treatment	1.430	0.608–3.363	0.413
Combination with colistin	0.783	0.178–3.444	0.746
Model D			
With skin and soft tissue infection	2.035	0.639–6.485	0.230
MDR Acb complex with TG or SB resistance	0.428	0.165–1.111	0.081
Bilateral pneumonia	2.711	1.013–7.254	0.047
Tigecycline-based treatment	1.362	0.598–3.102	0.462
Polymicrobial pneumonia	1.109	0.426–2.884	0.833
Model E			
With skin and soft tissue infection	1.979	0.612–6.405	0.254
MDR Acb complex with TG or SB resistance	0.422	0.160–1.110	0.080
Bilateral pneumonia	2.781	1.014–7.624	0.047
Tigecycline-based treatment	1.422	0.498–4.056	0.510
Polymicrobial pneumonia	1.086	0.413–2.853	0.868
Combination with colistin	0.797	0.179–3.557	0.767
Combination with carbapenem	1.006	0.346–2.921	0.991

*Abbreviations*: *MDR Acb* multidrug resistant *Acinetobacter calcoaceticus-Acinetobacter baumannii*, *TG* tigecycline, *SB* sulbactam

^a^No significant predictor was revealed when model A included polymicrobial pneumonia

^b^Bilateral pneumonia was the only significant predictor when model B included polymicrobial pneumonia (*p* = 0.049, adjusted odds ratio, 2.709; 95 % confidential interval, 1.004–7.305)

^c^Bilateral pneumonia was the only significant predictor when model C included polymicrobial pneumonia (*p* = 0.045, adjusted odds ratio, 2.783; 95 % confidential interval, 1.023–7.569)

### Monotherapy of tigecycline and sulbactam

Thirty-nine (46.4 %) patients had tigecycline monotherapy and 13 (15.5 %) had sulbactam or ampicillin/sulbactam monotherapy. Tigecycline group had significant lower rates of ventilator use, bilateral pneumonia, and airway eradication of MDR Acb complex. Both groups had similar clinical resolution rates. However, tigecycline group had lower rates of 30-day mortality and mortality during treatment (25.6 % versus 53.8 %, 17.9 % versus 30.8 %, *P* >0.05). In the univariate analysis for the patients with monotherapy, both the survivors and the deceased during treatment had no significant difference in demography, concomitant diseases, clinical conditions, and treatment (Table 4).

**Table 4:** The comparison and outcome analyses of the patients with monotherapy of tigecycline or sulbactam

Variables	TG group^a^	SB group^a^	*p*	Deceased^ab^	Survivors^a^	*p*
	*n* = 39	*n* = 13		*n* = 11	*n* = 41	
Demographic parameters						
Age, yr	71.4 (15.0)	68.7 (19.9)	0.899	75.3 (12.0)	69.5 (17.1)	0.439
Male gender	25 (64.1)	10 (76.9)	0.506	8 (72.7)	27 (65.9)	1.000
Concomitant diseases						
Hepatic dysfunction	3 (7.7)	0 (0.0)	0.564	1 (9.1)	2 (4.9)	0.518
Renal insufficiency	10 (25.6)	4 (30.8)	0.729	1 (9.1)	13 (31.7)	0.251
Chronic pulmonary disease	11 (28.2)	4 (30.8)	1.000	4 (36.4)	11 (26.8)	0.709
Heart disease	6 (15.4)	1 (7.7)	0.664	2 (18.2)	5 (12.2)	0.630
Diabetes mellitus	9 (23.1)	4 (30.8)	0.714	3 (27.3)	10 (24.4)	1.000
Immune compromise	8 (20.5)	1 (7.7)	0.420	2 (18.2)	7 (17.1)	1.000
Malignancy	8 (20.5)	3 (23.1)	1.000	4 (36.4)	7 (17.1)	0.216
Surgery	9 (23.1)	1 (7.7)	0.419	2 (18.2)	8 (19.5)	1.000
Clinical conditions						
APACHE II Score	17.0 (6.1)	18.2 (6.0)	0.557	17.1 (6.8)	17.3 (5.9)	0.904
Ventilator use	21 (53.8)	11 (84.6)	0.048	6 (54.5)	26 (63.4)	0.730
Bilateral pneumonia	18 (46.2)	12 (92.3)	0.004	8 (72.7)	22 (53.7)	0.319
Polymicrobial pneumonia	31 (79.5)	9 (69.2)	0.466	9 (81.8)	31 (75.6)	1.000
With MRSA	20 (51.3)	6 (46.2)	0.749	5 (45.5)	21 (51.2)	0.734
With *Pseudomonas aeruginosa*	14 (35.9)	3 (23.1)	0.506	5 (45.5)	12 (29.3)	0.470
With *Klebsiella* spp.	7 (17.9)	1 (7.7)	0.662	1 (9.1)	7 (17.1)	1.000
With *Serratia marcescens*	5 (12.8)	1 (7.7)	1.000	1 (9.1)	5 (12.2)	1.000
With *Stenotrophomanas maltophilia*	3 (7.7)	3 (23.1)	0.157	1 (9.1)	5 (12.2)	1.000
Multisite infections	14 (35.9)	5 (38.5)	1.000	4 (36.4)	15 (36.6)	1.000
With urinary tract infection	7 (17.9)	3 (23.1)	0.697	2 (18.2)	8 (19.5)	1.000
With catheter related infection	0 (0.0)	2 (15.4)	0.059	1 (9.1)	1 (2.4)	0.382
With skin and soft tissue infection	3 (7.7)	1 (7.7)	1.000	1 (9.1)	3 (7.3)	1.000
With intra-abdominal infection	4 (10.3)	0 (0.0)	0.561	0 (0.0)	4 (9.8)	0.567
With invasive fungal infection	6 (15.4)	0 (0.0)	0.317	1 (9.1)	5 (12.2)	1.000
Microbiology						
MDR Acb complex with TG or SB resistance^c^	6 (15.4)	4 (30.8)	0.244	1 (9.1)	9 (22.0)	0.668
Airway eradication of MDR Acb complex^d^	12 (34.3)	8 (88.9)	0.006	4 (57.1)	16 (43.2)	0.684
Bacteremia during treatment	2 (5.1)	0 (0.0)	1.000	1 (9.1)	1 (2.4)	0.382
Treatment						
Tigecycline-based treatment				7 (63.6)	32 (78.0)	0.435
Duration, days	13.8 (5.1)	12.7 (5.6)	0.293	11.9 (3.0)	14.0 (5.6)	0.398
Delayed treatment	20 (51.3)	5 (38.5)	0.423	5 (45.5)	20 (48.8)	0.845
Outcomes						
Clinical resolution of pneumonia	26 (66.7)	8 (61.5)	0.747			
Mortality during treatment	7 (17.9)	4 (30.8)	0.435			
30-day mortality	10 (25.6)	7 (53.8)	0.089			

*Abbreviations*: *TG* tigecycline, *SB* sulbactam, *APACHE* acute physiology and chronic health evaluation, *MRSA* methicillin resistant *Staphylococcus aureus*, *MDR Acb* multidrug resistant *Acinetobacter calcoaceticus-Acinetobacter baumannii*

^a^Categorical data are no.(%) of subject, continuous data are expressed as mean (standard deviation)

^b^Mortality during treatment

^c^The initial airway MDR Acb complex isolates with resistance to TG in TG group, or with resistance to SB in SB group

^d^35 patients in TG group and 9 in SB group; 7 patients in the deceased group and 37 in the survivors group had available data for evaluation

## Discussion

Prior case series studies reported clinical response rates ranging from 60 to 88 % in tigecycline treatment [9–11], and 67 to 75 % in sulbactam or ampicillin/sulbactam treatment for pneumonia involving MDRAB or MDR Acb complex [13–15]. There were only a few comparative studies investigating the efficacy of tigecycline or sulbactam, and usually they were compared with colistin or polymyxin, the other major treatment option for MDR Acb complex. Betrosian AP et al. reported that high-dose ampicillin/sulbactam monotherapy and colistin were comparably safe and effective treatment for critically ill patients with MDRAB VAP. The clinical success and improvement rate was 76.9 % for ampcillin/sulbactam group and 73.3 % for colistin group [22]. Oliveira MS et al. reported another study comparing ampicillin/sulbactam with polymyxins in treating infections caused by carbapenem-resistant *Acinetobacter* spp. [23]. In the study, about half of the enrolled patients had *Acinetobacter* bacteremia, and quarter of them had pneumonia. The mortality rate during treatment was 33 % in ampicillin/sulbactam group and 50 % in polymyxin group, and polymyxin use was an independent factor associated with mortality during treatment [23]. Chuang YC et al. reported a study comparing tigecycline-based to colistin-based therapy for MDRAB pneumonia in intensive care units. The tigecycline group has an excess mortality of 16.7 % (60.7 % versus 44 %, *P* = 0.04). The excess mortality of tigecycline is significant only among those with minimal inhibitory concentration (MIC) > 2 μg/mL, but not for those with MIC ≦ 2 μg/mL [24].

To our knowledge, the study was the first comparative study of tigecycline-based versus sulbactam or ampicillin/sulbactam-based treatment for pneumonia involving MDR Acb complex. Our two patient groups were from different but successive time periods, and the major treatment for MDR Acb complex was different in each time period in our hospital. Before August 2007, sulbactam or ampicillin/sulbactam was the only option probably with in vitro activity against MDR Acb complex in our hospital. After that, tigecycline became the major treatment option because of its high susceptibility rate to MDR Acb complex. However, the clinical and microbiological diagnostic criteria and definition, and standards of care and infection control were similar in both time periods. Covariate adjustment with multivariate analyses and matching with disease severity were performed to reduce bias of the historically controlled comparison.

The patients from both groups were aged with complicated underlying diseases and high disease severity. Higher rates of ventilator use and bilateral pneumonia reflected that sulbactam group might have a higher severity of pneumonia than tigecycline group. A higher rate of delay use in tigecycline group might reflect the early policy of tigecycline use in our hospital: usually tigecycline was not used as empiric or first-line regimen for nosocomial infection. Both groups had similar clinical outcomes. Bilateral pneumonia was the only independent predictor for mortality during treatment in different models of multivariate analysis. Combination therapy did not stand out as an independent predictor, which might be due to difference of combination strategies and regimens between the two patient groups. Most patients in sulbactam group had combination with carbapenem for synergistic effect against MDR Acb complex; however, tigecycline group mainly had anti-pseudomonal cephalosporins and fluoquinolones to cover *Pseudomonas aeruginosa*.

Because most patients in sulbactam group had concurrent carbapenem use, the study results in them might mainly reflect the efficacy of combination of sulbactam and carbapenem. In the comparative analyses of monotherapy, the patients with sulbactam or ampicillin/sulbactam monotherapy had relatively higher mortality rates than the patients with tigecycline monotherapy or the overall sulbactam group. The results implied that combination with carbapenem might improve clinical outcomes of sulbactam-based treatment. Besides, more than half of the sulbactam group had sulbactam-resistant MDR Acb complex isolates during treatment. Combination with carbapenem might play a role giving a high airway eradication rate. The patients with sulbactam monotherapy also had a high airway eradication rate, but most of them did not have sulbactam-resistant MDR Acb complex.

Synergistic effect against MDRAB with the combination of sulbactam and carbapenem had been reported [4, 25]. However, the synergistic effect was associated with the MICs of carbapenem and sulbactam. If the MICs exceeded achievable serum levels, the potential of sulbactam/carbapenem combination as treatment regimen for MDRAB infections might be limited [4]. In our study, full or intermediate sulbactam resistance was detected in 84.5 % of tigecycline-treated patients, therefore, physicians tended to use tigecycline for these patients with sulbactam-resistant MDB Acb complex. For these cases, the clinical outcomes of sulbactam group might not be achieved if they received sulbactam/carbapenem combination therapy.

There are some other limitations in this study. First, our respiratory specimens were clinical specimens from clinical practice, and they might not be obtained from deep sites in the lungs. Growths of etiologic pathogens were assessed semi-quantitatively if the specimens were qualified for culture. We cannot absolutely distinguish airway MDR Acb complex infections from colonization. However, our definition for pneumonia was practical, and our conclusion based on clinically relevant data and management could provide important information for clinical practice. Second, polymicrobial pneumonia and concomitant infections were common, and the clinical impact of other etiologic pathogens or extrapulmonary infections was not evaluated comprehensively. Third, we studied MDR Acb complex rather than MDRAB. Although prior studies reported that about 90 % of Acb complex with multidrug or carbapenem resistance was the genomic specie of *A.baumannii*, comparison with studies on *A. baumannii* isolates are not straightforward [26].

## Conclusions

Tigecycline-based treatment had a similar clinical outcome to sulbactam or ampicillin/sulbactam-based treatment for pneumonia involving MDR Acb commplex, but tigecycline group had a lower microbiological eradiation rate. More comparison studies are essential to establish the optimal regimens for pneumonia involving MDR Acb complex.

## Abbreviations

AB, *Acinetobacter baumannii*; Acb, *Acinetobacter calcoaceticus-Acinetobacter baumannii*; APACHE, acute physiology and chronic health evaluation; CGMH, Chang Gung Memorial Hospital; CI, confidence interval; MDR, multidrug-resistant; MIC, minimal inhibitory concentration; OR, odds ratio; VAP, ventilator associated pneumonia
